# Fracture resistance and marginal gap formation of post-core restorations: influence of different fiber-reinforced composites

**DOI:** 10.1007/s00784-019-02902-3

**Published:** 2019-05-16

**Authors:** Márk Fráter, Lippo Lassila, Gábor Braunitzer, Pekka K. Vallittu, Sufyan Garoushi

**Affiliations:** 1 Department of Operative and Esthetic Dentistry, Faculty of Dentistry, University of Szeged, Szeged, Hungary; 2 Department of Biomaterials Science and Turku Clinical Biomaterials Center - TCBC Institute of Dentistry, University of Turku, Itäinen Pitkäkatu 4 B, FI-20520 Turku, Finland; 3 dicomLAB Dental Ltd., Szeged, Hungary; 4 City of Turku Welfare Division, Oral Health Care, Turku, Finland

**Keywords:** Fracture load, Post-core material, Short fiber composite, Microgap

## Abstract

**Objectives:**

The aim was to explore the fracture behavior and marginal gap within the root canal of endodontically treated (ET) premolars restored with different fiber-reinforced post-core composites (FRCs). Further aim was to evaluate the composite curing at different depths in the canal.

**Materials and methods:**

Eighty-seven intact upper premolars were collected and randomly divided into six groups. After endodontic procedure, standard MOD cavities were prepared and restored with their respective fiber-reinforced post-core materials: group 1: prefabricated unidirectional FRC-post + conventional composite core; group 2: prefabricated unidirectional FRC-post + short fiber composite (SFRC) core; group 3: individually formed unidirectional FRC-post + conventional composite core; group 4: randomly oriented SFRC directly layered as post and core; group 5: individually formed unidirectional FRC + randomly oriented SFRC as post and core. After restorations were completed, teeth (*n* = 3/group) were sectioned and then stained. Specimens were viewed under a stereo microscope and the percentage of microgaps within the root canal was calculated. Fracture load was measured using universal testing machine.

**Results:**

SFRC application in the root canal (groups 4 and 5) showed significantly higher fracture load (876.7 N) compared to the other tested groups (512–613 N) (*p* < 0.05). Post/core restorations made from prefabricated FRC-post (group 1) exhibited the highest number of microgaps (35.1%) at the examined interphase in the root canal.

**Conclusions:**

The restoration of ET premolars with the use of SFRC as post-core material displayed promising performance in matter of microgap and load-bearing capacity.

**Clinical significance:**

Fracture resistance of ET premolar restored by bilayered composite restoration that includes both SFRC as post-core material and surface conventional resin seems to be beneficial.

## Introduction

Endodontically treated (ET) teeth are structurally different from non-restored vital teeth and require special restorative treatment [1]. The loss of structural integrity is the main reason why ET teeth are vulnerable and show reduced resistance to fracture [2, 3]. This is due to previous caries and excessive removal of dentine during root canal treatment, rather than low moisture content or increased brittleness [4, 5]. As a consequence, root-filled teeth are at an increased risk of fracture [4, 6]. This is especially important in the case of ET premolars, as numerous studies report a high fracture incidence for these teeth, mainly the maxillary ones [7–9]. Maxillary premolars are exposed to a combination of shearing and compressive forces, which makes them especially prone to fracture [10]. The loss of marginal ridges makes this even more pronounced. Conservative endodontic access cavity preparation in posterior teeth reduced the relative cuspal stiffness by only 5 to 20% [11, 12]. At the same time, standardized mesial-occlusal-distal (MOD) cavity preparation in maxillary premolars resulted in an average loss of 63% in relative cuspal stiffness [13], which is related mainly to the loss of marginal ridge integrity [14]. This has been confirmed by Wu et al. who observed a dramatic increase in cuspal deflection as a result of the removal of both marginal ridges in an MOD cavity preparation and in conjunction with an endodontic access cavity [15].

Furthermore, it has been pointed out that endodontic treatment and extensive restorative procedures (e.g., MOD cavity preparation) combined with high occlusal loads and lateral excursive contacts lead to higher susceptibility to fracture [10, 16], which poses a threat to maxillary premolars. Therefore, an adequate restorative approach must fulfill both esthetics and the structural preservation and reinforcement of these teeth, so that they are protected against fracture.

The use of fiber-reinforced composite (FRC) posts has become very popular to restore ET teeth, due to their favorable modulus of elasticity which is closer to that of dentine compared to metal posts [17, 18]. Several studies showed that inserting a post into ET premolars significantly increased their fracture resistance [19–21]; however, other studies only managed to prove the positive effect of post placement on the fracture pattern of such premolar teeth [22, 23]. The latter was also confirmed by Trope et al. [24] and Zicari et al. [25] who concluded that the application of an FRC post does not actually strengthen the given tooth. Contrary to that, in a recent study of ours, we found that the application of multiple posts instead of a single post, especially when using multiple an individually formed FRC posts, lead to better reinforcement and stress transfer [26]. In fact, many authors also showed in their in vitro studies that endodontically treated teeth restored with an individually formed fiber post exhibited significantly higher fracture resistance than those restored with a single prefabricated fiber post [27, 28].

In 2007, Garoushi and co-workers found that the restoration of anterior ET teeth with short fiber-reinforced composite (SFRC) yielded a better load-bearing capacity as opposed to the application of an FRC post [29]. This was partly confirmed by Forster et al. in ET premolar teeth with class I cavity. In that study, the directly layered fiber-reinforced composite post and core (DLFRC) group showed statistically non-significant difference compared to intact premolar teeth in terms of fracture resistance [30].

Based on this knowledge, it is important to obtain more detailed information on this fiber-reinforced post-core restorative approach. Thus, the aim of the present investigation was to compare the load-bearing capacity of various fiber-reinforced post and core direct restorative methods for the reinforcement of ET premolar teeth with MOD cavities. Also, the curing performance at different depths and adaptation of materials within the root canal for each method were investigated. The null hypotheses were that (1) there would be no difference in the maximal fracture load or in fracture pattern between the tested groups and (2) there would be no difference in the curing performance or the marginal microgap within the root canal of the ET teeth restored with the study methods.

## Materials and methods

All procedures of the study were approved by the Ethics Committee of the University of Szeged, and the study was designed in accordance with the Declaration of Helsinki.

Eighty-seven upper premolar teeth, extracted for periodontal or orthodontic reasons, were selected for this investigation. The freshly extracted teeth were immediately placed in 5.25% NaOCl for 5 min and stored in 0.9% saline solution at room temperature. Teeth were used within 2 months after extraction. During specimen preparation, the soft tissue covering the root surface was removed with hand scalers. The inclusion criteria were absence of caries or root cracks, the absence of previous endodontic treatments, posts or crowns, resorptions, or evident lateral canals. Buccolingual and mesiodistal radiographs of all teeth were taken and examined to evaluate root integrity and the number of canals present. To standardize procedures and materials, all teeth used in this study had one root canal with a curvature of less than 5°, evaluated by Schneider’s technique [31], and teeth with a root length of 15 ± 1 mm and similar mesiodistal and buccolingual dimensions (± 10%) were selected. Ninety percent of the specimen ranged 9–10 mm in size, measured at the widest buccolingual dimension, and the rest measured were 6.5–8 mm. Regarding the mesiodistal dimension, 90% of the specimen ranged 7–7.5 mm, and the rest were 6.5–8 mm.

The teeth were randomly distributed over six study groups of 15 specimens each, with one group only containing 12 specimens. The teeth in this later group were left intact to serve as control (group 6, *n* = 12). MOD cavity preparation and later on root canal treatment were performed by the same trained operator in the rest of the groups (groups 1–5).

### Specimen preparation

A standardized mesio-occlusal-distal (MOD) cavity was prepared on teeth using a round-end parallel diamond (881.31.014 FG - Brasseler USA Dental, Savannah, GA) with water coolant so that the buccopalatal width of the occlusal isthmus was one third of the intercuspal width, and the proximal box width was half of the buccopalatal width of the crown. The gingival floor was located 1 mm above the cemento-enamel junction (CEJ). All internal angles were rounded and the cavosurface margins were at 90°. After finalizing the MOD cavity preparation, access cavity preparation was carried out with a round-end diamond bur (850–014 M SSWhite, Lakewood, NJ, USA) with water cooling and root canal treatment was performed in the prepared teeth. The working length was established with the direct method by subtracting 1 mm from the real root length determined by introducing a number 10 K-file (Maillefer-Dentsply, Ballaigues, Switzerland) until it was visible through the apical foramen. The canals were instrumented using rotary ProTaper Universal files (Dentsply, Maillefer, Ballaigues, Switzerland). The ProTaper sequence (S1, S2, F1, F2) was used for the preparation at the working length. Irrigation was performed after every instrument with 2 ml of 2.5% NaOCl solution and the canal space was filled with irrigant during the instrumentation phase. After the shaping and cleaning of the root canal, the roots were dried with 96% alcohol and paper points. Root canal filling was done by matched single-cone obturation with a master cone (F2 gutta-percha, Maillefer-Dentsply, Ballaigues, Switzerland) and sealer (AH plus; Dentsply De Trey GmbH, Konstanz, Germany). The access cavity was temporarily filled with Fuji Triage Pink (GC Europe, Leuven, Belgium). Fuji Triage Pink was applied to the apical part of the root in order to prevent leakage through the apex. The teeth were stored in an incubator (mco-18aic, Sanyo, Japan) for 1 week (at 37 °C, 100% relative humidity).

All root canal-treated teeth received a minimal invasive post space preparation with a depth of 7–8 mm, as measured from the CEJ on the buccal aspect of the tooth, but no post preparation drills recommended by the manufacturer were used in order to preserve the individual anatomy of the specimen teeth. Only the root canal filling was removed with Number 3 Gates Glidden burs and ISO standard Hedstrom files leaving a minimum apical seal of 4–6 mm of gutta-percha in the canal. The Number 3 Gates Glidden bur was used on the full 7–8 mm length.

After cutting back the gutta-percha, the root canal was rinsed with chlorhexidine and dried with paper points.

In group 3 after drying the canal, an individualized FRC post was fabricated directly in the canal. The root canal received as many 0.9-mm-sized uncured FRC posts (everStick POST, GC Europe, Leuven) as possible bundled according to the thickness of the root canal using the lateral condensation method described by Hatta et al. [32]. These posts were gently removed as one unit with a needle-nose plier from the root canal and then light cured for 40 s. It was confirmed in all cases that the individualized posts were repositioned to their original position into the canal after light curing.

All teeth received the same adhesive treatment. Prior to the adhesive treatment of the cavity and the root canal, a Tofflemire (1101C 0.035, Hawe-Neos, Italy) matrix band was applied, and the enamel was acid-etched selectively with 37% phosphoric acid for 15 s and rinsed with water. The root canal and the coronal cavity were rinsed with 2 ml of water and dried with paper points and air. For bonding, a dual-cure one-step self-etch adhesive system (Gradia Core Self-Etching Bond, GC Europe, Leuven, Belgium) was used, according to the manufacturer’s instructions using a microbrush-X disposable applicator (Pentron Clinical Technologies, LLC, USA). Excess adhesive was removed by suction drying (Evacuation Tip – Starryshine, Anaheim, CA, USA) within 0.5 cm from the occlusal cavity (without contact). Excess adhesive resin at the bottom of the canal was removed with a paper point. The adhesive was light cured for 60 s using an Optilux 501 quartz-tungsten-halogen light-curing unit (Kerr Corp., Orange, CA, USA). The average power density of the light source, measured with a digital radiometer (Jetlite light tester; J. Morita USA Inc. Irvine, CA, USA) prior to the bonding procedure, was 840 ± 26.8 mW/cm^2^. After light curing the adhesive, the interproximal walls were build up with composite (G-aenial Posterior PJ-E, GC Europe, Leuven, Belgium) using the centripetal technique, thus transforming the MOD cavity into a class I cavity. Each interproximal wall was light cured for 40 s.

**Fig. 1:**
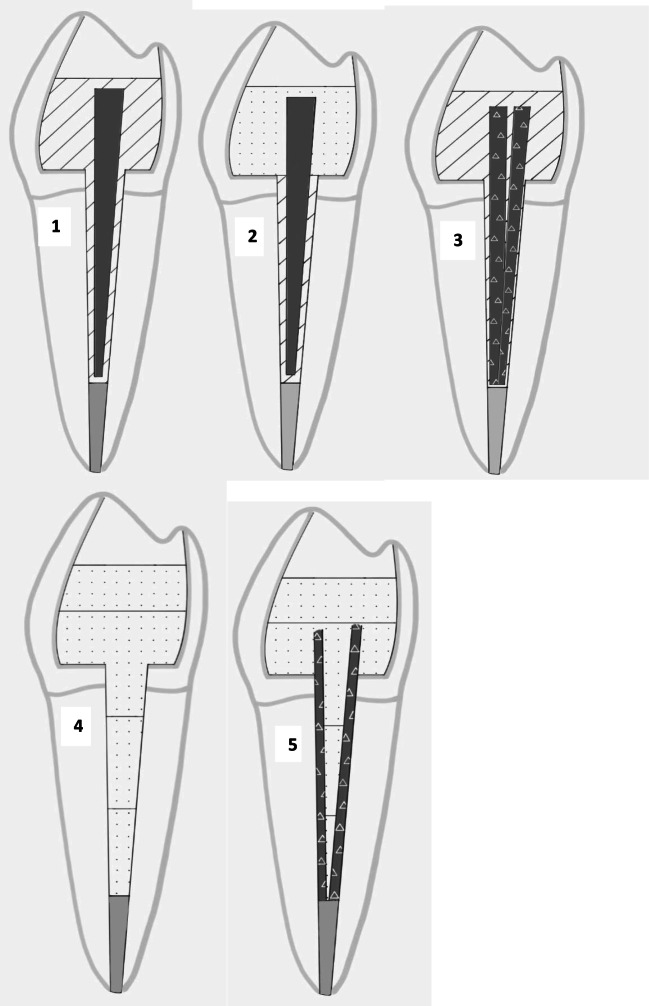
Schematic figure representing the test groups. Group 1: prefabricated FRC post + conventional composite core; group 2: prefabricated FRC post + SFRC core; group 3: individually formed FRC post + conventional composite core; group 4: SFRC directly layered as post and core; group 5: individually formed FRC + SFRC as post and core

Five different techniques were used to restore the specimens in groups 1–5. (Fig. 1):

- Group 1

The teeth received a prefabricated, conventional continuous unidirectional glass FRC post (GC Fiber post, GC Europe, Leuven, Belgium). Before the adhesive treatment, the conventional translucent FRC posts of 0.8 mm diameter (GC Fiber Post, GC Europe, Leuven, Belgium) were tried in and cut to a length 1 mm below the level of the occlusal cavity margins with a water-cooled diamond disc (Isomet 2000; Buehler Ltd., Lake Bluff, IL, USA) and cleaned with alcohol after try in. The posts received silanization of the surface (Ceramic Primer, GC Europe, Leuven, Belgium) following the manufacturer’s recommendation. After silanization, the post surface was bonded with the same bonding agent used for the cavity. Luting of the posts and the core buildup was performed with a dual-cure resin composite core material (Gradia Core, GC Europe, Leuven, Belgium). Gradia Core was applied using its own automix cartridge with an “elongation tip” for direct root canal application. After insertion of the post, 5 min of chemopolymerization time was provided to reduce polymerization stress, then cement was light cured for 40 s from each side (a total of 160 s/tooth). The outlines of the restoration were finished with dental composite (G-aenial Posterior P-JE, GC Europe, Leuven, Belgium), which was light cured for 40 s. This final step is the same in all restored groups.

- Group 2

The teeth received a prefabricated, conventional continuous unidirectional glass FRC post (GC Fiber post, GC Europe, Leuven, Belgium). The post was adhesively treated and luted the same way into the root canal as described in group 1. The core buildup around the post for restoring the coronal cavity was performed with randomly oriented short glass fiber-reinforced composite (SFRC) (EverX Posterior, GC Europe, Leuven, Belgium) packed around the post using approximately 3-mm-thick increments in a horizontal manner. Each increment was light cured from the occlusal surface for 40 s. The last 1-mm-thick occlusal layer was conventional particulate-filled composite (G-aenial Posterior) covering the SFRC.

- Group 3

Teeth received an individualized FRC post formed from 2 to 3 pieces of FRC posts as previously described. These posts did not receive any surface treatment in accordance with manufacturer’s instructions. Luting of the individualized posts and the core buildup was performed with a dual-cure resin composite core material (Gradia Core, GC Europe, Leuven, Belgium) the same way as in group 1. The outlines of the restoration were finished with conventional composite material.

- Group 4

The teeth were reconstructed with the method described by Forster et al. [30] building a direct layered FRC post and core from SFRC. The original protocol was slightly modified as here the FRC post and core was horizontally layered in 3–4 mm segments. An increment of SFRC was packed to the apical portion of the postspace using a microbrush-X disposable applicator (Pentron Clinical Technologies, LLC, USA). A light-transmitting FRC post (0.8 mm GC Fiber post, GC Europe, Leuven, Belgium) was inserted into the postspace in order to aid the transmission of the light to the apically positioned layers. The “light-transmitting” post was withdrawn with 0.5–1 mm from the surface of the uncured SFRC layer not to have direct contact with it. After each layer, 80 s of light curing through the fiber post was carried out. After incrementally filling the root canal to the level of the CEJ with repeating the previously described procedure, SFRC was layered in the coronal cavity until 1 mm below the margin of the occlusal cavity in a concave shape. Each coronally placed increment was light cured from the occlusal surface for 40 s. The last 1-mm-thick occlusal layer was conventional composite material covering the SFRC.

- Group 5

One 0.9-mm-sized uncured post (everStick POST, GC Europe, Leuven) was cut longitudinally with a sharp straight scissor and applied on the buccal and lingual walls of the root canal. Once tight contact was achieved, the posts were light cured for 40 s. The posts were also slightly extending into the coronal cavity. The space remaining between the posts in the root canal and later the coronal cavity was filled up with SFRC described in group 4. The outlines of the restoration were finished with conventional composite material.

Finally, for all restored teeth, glycerine gel (DeOx Gel, Ultradent Products Inc., Orange, CA, USA) was applied and final polymerization from each side for 40 s was performed. The restorations were finished with a fine granular diamond burr (FG 7406-018, Jet Diamonds, USA and FG 249-F012, Horico, Germany) and aluminum oxide polishers (OneGloss PS Midi, Shofu Dental GmbH, Ratingen, Germany).

### Mechanical loading test

After the restorative procedures, the specimens were stored in physiological saline solution (Isotonic Saline Solution 0.9% B. Braun, Melsungen, Germany) in an incubator (mco-18aic, Sanyo, Japan) for 1 week (at 37 °C, 100% humidity) before the fracture loading test. Prior to embedding, the root surface of each tooth was coated with a layer of liquid latex separating material (Ruber-Sep, Kerr, Orange, CA, USA) to simulate the periodontal ligament. Specimens were embedded in methacrylate resin (Technovit 4004, Heraeus-Kulzer) at 2 mm from the CEJ to simulate the bone level. After embedding, all specimens were immediately subjected to a static loading test using a universal loading device (5848 MicroTester1, Instron, Norwood, MA, USA). Each test was performed at a cross-head speed of 0.5 mm/min and load was applied at 45° using a 4.8-mm-diameter stainless steel ball-shaped stylus positioned to the central groove of the tooth providing two contacts with the triangular ridges and one with the more dominant marginal ridge. The maximum failure load was recorded in newtons (N). A force vs. extension curve was dynamically plotted for each tooth. After mechanical testing, the specimens were examined for fracture patterns. According to Scotti and co-workers, distinction was made between restorable or nonrestorable fractures under optical microscope with a two-examiner agreement. A restorable fracture is above the CEJ, meaning that in case of fracture, the tooth can be restored, while a nonrestorable fracture extends below the CEJ and the tooth is likely to be extracted [33].

### Microgap determination test

Five groups, each consisting of 3 endodontically treated and restored teeth, were investigated in the microgap determination test. The teeth (*n* = 15) were restored in the same way as mentioned earlier. Teeth were sectioned mid-sagitally in the mesiodistal plane using a ceramic cutting disc operating at a speed of 100 rpm (Struers, Glasgow, Scotland) under water cooling. In each group, one of the sectioned restoration that contains the post was further ground and polished using #4000-grit silicon carbide papers at 300 rpm under water cooling using an automatic grinding machine (Rotopol-1; Struers, Copenhagen, Denmark). Then, sectioned teeth were painted with permanent marker and polished gently for few seconds. The dye penetration along post/core margins of each section was evaluated independently using a stereo microscope (Heerbrugg M3Z, Heerbrugg, Switzerland) at a magnification of × 6.5 and the extent of dye penetration was recorded in mm as a percentage of the total margin length [34].

### Microhardness test

Microhardness of luting composite inside the canal was measured using a Struers Duramin hardness microscope (Struers, Copenhagen, Denmark) with a 40 objective lens and a load of 1.96 N applied for 10 s. Each sectioned restoration was subjected to 5 indentations on the top (coronal part) and the bottom (apical part) of the canal. The diagonal length impressions were measured and Vickers values were converted into microhardness values by the machine. Microhardness was obtained using the following equation:

$$ H=\frac{1854.4\times P}{d^2} $$

where *H* is Vickers hardness in kg/mm^2^, *P* is the load in grams, and *d* is the length of the diagonals in μm.

### Statistical analysis

The data were statistically analyzed with SPSS version 23 (SPSS, IBM Corp.) using analysis of variance (ANOVA) at the *p* < 0.05 significance level followed by a Tukey HSD post hoc test to determine the differences between the groups.

## Results

**Fig. 2:**
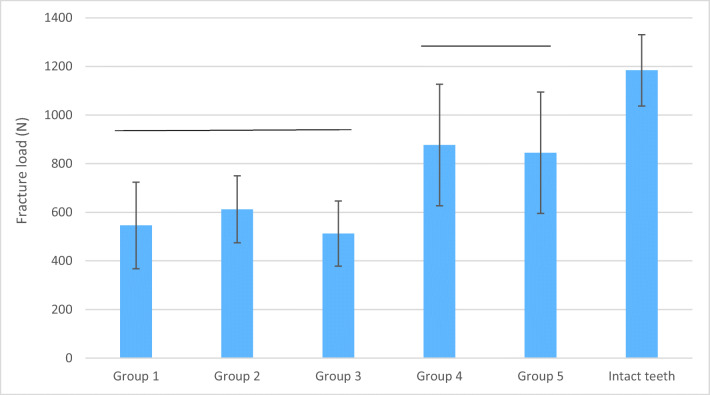
The mean values for the fracture loads (N) and standard deviation of the restored teeth (SD). Horizontal lines above the columns indicate groups that do not differ statistically from each other

Figure 2 summarizes the fracture load for the different study groups. The control group (intact teeth) showed the highest fracture load (1183.9 N) and was significantly better compared to all restored groups (*p* < 0.05). The application of SFRC in the root canal (groups 4 and 5) showed significantly higher fracture resistance (876.7 N) compared to the other tested groups (groups 1, 2, and 3) (*p* < 0.05). There was no statistically significant difference (*p* > 0.05) between the groups using SFRC inside the canal (groups 4 and 5). Therefore, the null hypothesis regarding fracture load was rejected.

Regarding fracture pattern, all restored groups using either SFRC or individually formed FRC posts, just as the control group, showed dominantly repairable fractures, whereas the group using conventional FRC post for reinforcement (group 1) showed dominantly unrepairable fractures (Table 1). Therefore, the null hypothesis regarding fracture patterns was also rejected.

**Table 1:** The distribution of fracture pattern among the study group

Fracture pattern	Group 1	Group 2	Group 3	Group 4	Group 5	Intact teeth
Restorable	3	7	7	8	9	10
Nonrestorable	9	5	5	4	3	2
Restorable %	25	58	58	67	75	83
Non-restorable %	75	42	42	33	25	17

**Fig. 3:**
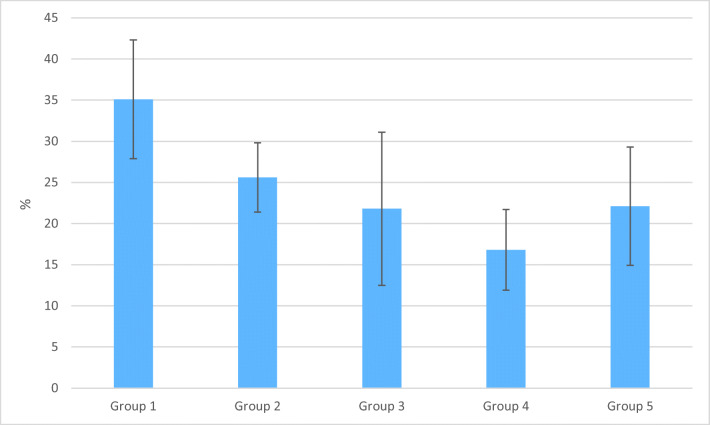
Mean percentage of microgap observed in different groups from total post/core-tooth interface length after staining. Vertical lines represent standard deviation

The mean values and standard deviations of microgap percentage at post/core-tooth interface of the five groups are presented in Fig. 3. Data showed that post/core restorations made from directly layered SFRC (group 4) had a lower microgap (16.8%) than other groups, whereas group 1 exhibited the highest number of microgap (35.1%) at the examined interphase in the root canal (Fig. 3).

**Fig. 4:**
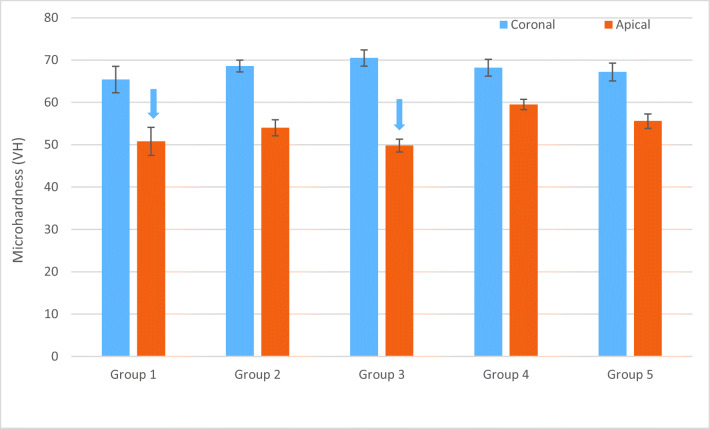
Microhardness (VH) mean values for resin composites at the top (coronal) and bottom (apical) parts of the root canal. Arrows above the columns indicate VH of these groups dropped below 80% of the coronal part values. Vertical lines represent standard deviation

In terms of the luting composite’s microhardness within the apical part of the canal, group 4 produced the highest microhardness values (59.5 VH) and also the smallest difference between the microhardness measured at the apical and at the coronal part of the root canal (Fig. 4).

## Discussion

The quality and longevity of restorations in ET teeth play an important role in the outcome and must be considered as a critical final step for successful endodontic therapy [35]. The ideal rehabilitation of ET posterior teeth would improve their mechanical resistance and prevent unfavorable fractures, thereby restoring anatomy and function [36]. In this study, maxillary ET premolars with MOD cavities were used as they present an unfavorable anatomy in crown volume and crown-to-root proportion, which makes them more susceptible to cusp fractures than other posterior teeth when exposed to occlusal load [37]. The presence of an MOD cavity configuration might lead to a further major biomechanical problem. According to Hood’s hypothesis, cusps of teeth with MOD cavity preparations function as a cantilever beam, with the extent of deflection under load influenced by both beam thickness and length [38], meaning the prepared cavity floor serves as a fulcrum for cusp bending and the cantilever length increases with the cavity depth [39].

In everyday clinical practice, direct tooth-colored restorations are often used for ET teeth as a relatively low cost, esthetic alternative to cuspal coverage restorations [4]. However, insufficient material properties limit the success of direct composite restorations in high stress-bearing areas [40, 41]. Forster et al. demonstrated that once the depth of an MOD cavity reaches 5 mm, a direct filling using conventional composite material on its own cannot reinforce the damaged tooth anymore [42]. This is in accordance with Eapen et al. [43] and Kemaloglu et al. laboratory findings [44].

As a consequence, the majority of dental practitioners routinely restore root-filled maxillary premolars with fiber posts to reinforce them [7]. Yet, the results are controversial. Some studies claim fiber posts increase the resistance of ET premolar teeth, whereas others showed that fiber posts do not strengthen the teeth but reduce the incidence of catastrophic fractures [23, 24, 45]. In our study, the groups restored with conventional FRC post (groups 1 and 2) showed significantly lower fracture resistance than the groups with SFRC inside the root canal (groups 4 and 5), and conventional FRC reinforcement failed to restore close-to-control fracture resistance. Several authors have pointed out that the diminished reinforcing effect of FRC posts might be attributed to the removal of more tooth material during post placement, which possibly weakens the root [7, 26, 36, 46–48]. In our study, as previously described [30], minimally invasive method of post site preparation was applied in all restored groups to avoid this effect. Because of this, the smaller conventional FRC post could not fill out the root canal entirely, possibly leading to greater amount of luting composite in the available space. The mismatch between the diameter of the fiber post and that of the post site remains a well-known clinical challenge [49, 50]. If the post does not fit well, especially at the coronal level, the resin cement layer would be excessively thick, and bubbles are likely to form in it, which can lead to de-bonding [49, 51]. This might be one of the reasons behind the inferior performance of the conventional FRC posts. In group 1, the missing coronal dentine was replaced with the FRC post and the same composite core buildup material used to lute the post, which represents one of the easiest and most popular restorative solutions among the practitioners—and the primary recommendation of manufacturers of dual-cure core buildup materials. One of the main drawbacks of particulate-filled conventional composite and dual-cure core buildup materials when used to substitute the missing dentine is the significantly lower fracture toughness of these composite materials compared to that of the dentine [40]. In group 2, the missing coronal dentine was substituted with SFRC, beside the conventional FRC post. SFRC is a dental restorative composite intended to be used in high stress-bearing areas as a dentine replacing material [52–55]. Mechanical testing has shown major improvements in the load-bearing capacity, the flexural strength, and also the fracture toughness of SFRC in comparison with particulate-filled conventional composite materials. In our study, SFRC together with a conventional FRC post (group 2) did not yield significantly better results compared to the dual-cure core buildup material together with FRC post (group 1). This may be attributed to the poor adhesion between the conventional FRC post and any composite material. All FRC posts are made of two main components: the reinforcing fibers and the polymer matrix. Matrix polymers are generally epoxy resins or other thermosetting polymers with a high degree of conversion and a highly cross-linked structure [56, 57], which makes it very difficult to bond the prefabricated conventional FRC posts to any composite resin or to the tooth structure [58].

One possible solution to overcome the irregular root canal anatomy left by the minimal invasive post space preparation and intentional dentine preservation is to apply multiple posts in the same canal (multi-post technique) or to use an individual formed post. In group 3, an individualized FRC post formed from 2 to 3 pieces was used, as previously described by Hatta et al. [32]. Individually formed posts consist of continuous unidirectional E-glass fibers and a multiphase polymer matrix forming the semi-interpenetrating polymer network (semi-IPN). In the semi-IPN structure, there are both linear and cross-linked polymer phases. Due to the presence of the linear polymer phase, this material has shown good bonding between the post, the cement, and the dentine compared to the bonding of conventional FRC posts with a high cross-linked polymer matrix [28, 59, 60]. Although theoretically this individualized post should produce a better fit in the root canal, thus less cement could be used, group 3 was not superior to the single conventional FRC post groups (groups 1 and 2). This is in contrast with our previous findings [26], where any multi-post technique was significantly better than the single conventional FRC post buildup in terms of the achieved fracture resistance. It is obvious that this difference can be attributed to major differences in the study design: in our previous study, the teeth were decoronated, without any ferrule and without any definite coronal restoration.

Of the techniques tested in the present study, the Bioblock technique (group 4) was characterized by significantly higher fracture resistance than any of the other techniques utilizing FRC posts (groups 1, 2, and 3). This could be explained by examining the tooth from a biomechanical point of view. As pointed out by Le Bell-Rönnlöf et al., since the conventional FRC post is placed in the most central part of the post site (neutral axis of the tooth), the post is not optimally placed in terms of biomechanics if reinforcement is the desired outcome [28]. In fact, the surface of the post canal dentinal wall is a more appropriate choice for post placement for reinforcement, as this is where the highest tensile stresses occur [61]. With the Bioblock technique, SFRC is directly and closely adapted to the root canal wall, eliminating the drawbacks of the usage of luting cement or the “biomechanically incorrect” positioning of the FRC post, thus potentially eliminating all the damaging tensile stresses produced when the restoration is loaded. This is supported by other studies, suggesting that the survival rate of restorations might be increased if the fibers are placed at the interface [62, 63].

This concept is in accordance with the monoblock theory, which states that it is always beneficial to reduce the number of interphases as they do not only concentrate, but also increase the amount of stress inside a restoration [64]. According to the protocol of the present study, the thickness of each SFRC layer inside the tooth was increased from 2 mm [30] to 3–4 mm, as it has been proven that SFRC can be adequately light cured to 4–5 mm safely [65]. This is due to both the translucent nature of the material and the fact that the randomly oriented fibers within it may conduct and scatter the light over longer distances [66].

Still the question arises whether the SFRC material could have adequate curing also inside the root canal. Therefore, microhardness test was performed on the restorative techniques used within the root canal. The results showed that all restorative materials used in the coronal portion of the canal had higher microhardness compared to the apical portion of the same canal, indicating better curing due to higher intensity of light polymerization. In the critical apical portion, there was no difference in the microhardness of the used materials. This is interesting since in groups 4 and 5, SFRC was used inside the root canal, which needs light curing to set, while in the rest of the restored groups, a dual-cure core buildup material was used. This shows the efficiency of the curing protocol proposed by Forster et al. with the Bioblock technique using a conventional FRC post inside the canal just for light transmitting purposes [30]. The highest microhardness grades in the apical portion of the canal were achieved with group 5 using 2 pieces of individually formed posts together with SFRC inside the canal for reinforcement. In this hybrid technique, the individually formed posts were directly luted to the opposing root canal wall in order to act as a potential stress-absorbing layer as suggested by Vallittu et al. [67] and Le Bell Rönnlöf et al. [28]. Also the mean difference between the coronal and apical microhardness values was the lowest in group 5. This could be attributed to the potential light-transmitting capacity of the individually formed FRC posts inside the canal.

Since the adaptation of the used materials within the canal seems to be of key importance, gap formation was also evaluated with a microgap determining test for each technique. Microgap scores were the highest in group 1 and lowest in group 5. This is in accordance with the findings of Patel et al. [68]. The shrinkage stress and consequent gap formation when using dual-cure core buildup materials for luting in the root canal is a well-known problem due to the extremely high C-factor in this specific area [69, 70]. With the SFRC material, the control of the polymerization shrinkage stress is achieved by fiber orientation [54]. Therefore, during polymerization, the material is not able to shrink along the length of the fibers. It retains its original dimensions horizontally, but the polymer matrix between the fibers can shrink, leading to a better adaptation to the root canal walls (groups 4 and 5).

Regarding the fracture patterns, the restored groups produced predominantly favorable fracture patterns, except for group 1. Shifting the fracture pattern towards repairable fractures is a well-known phenomenon when using SFRC [44, 71, 72] as it acts as a stress-absorbing and crack-stopping layer, which can be explained by the size of the incorporated fibers. In order for a fiber to act as an effective reinforcement for polymers, stress transfer from the polymer matrix to the fibers is essential [73, 74]. This is achieved by having the fiber length equal or greater than the critical fiber length [54]. It has been measured that the critical fiber lengths of E-glass with bis-GMA polymer matrix vary between 0.5 and 1.6 mm [75]. SFRC fulfills this requirement with fiber lengths of 1 to 2 mm. Interestingly, in our study, it was not possible to reinforce the teeth to a satisfactory extent with the multi-post technique (group 3), but the fracture patterns were still mostly favorable. This might be caused by the number and unique features of these uncured posts, as described above. Group 1, with the conventional FRC post and dual-cure core buildup material, was characterized predominantly by unfavorable fractures. This is in line with the latest findings of Lazari et al. [76].

Although the application of SFRC (groups 4 and 5) yielded significantly better fracture resistance than any type or number of FRC posts (groups 1, 2, and 3), the achieved fracture resistance was still significantly lower than the fracture resistance of the control group (intact teeth). This might indicate the necessity of cuspal coverage in premolar ET teeth with MOD cavities when reinforcement is the primary aim.

The tested specimens received an oblique load (45° to the long axis of the tooth) which appears to be the worst-case scenario in terms of the fracture resistance of ET teeth as described by Wandscher et al. [77]. The limitation of this investigation is that static load to fracture test was used to determine maximal fracture resistance instead of applying cyclic loading. Stress applied to the teeth and dental restorations is generally low and repetitive rather than being isolated and impactive in nature. However, because of a linear relationship between fatigue and static loading, the compressive static test also gives valuable information concerning the fracture behavior and load-bearing capacity [78]. According to Taha et al., “In experimental studies, fracture resistance to static loading has been used as a measure of the effect of cavity preparation and/or restoration on tooth strength. Although the fracture load is typically much higher than functional occlusal loads, it is still a valid method for comparing restorative materials and different cavity designs.” [4]. Also, as stated by Le Bell-Rönnlöf et al., static loading is usually the first step in the evaluation process of a novel dental material and related technique and is commonly used in order to obtain basic knowledge regarding the fracture behavior and load capacity of a post restored tooth [28]. Given the mentioned shortcomings, the proposed techniques should require future testing with dynamic loading.

## Conclusions

The restoration of endodontically treated premolars with the use of SFRC as post-core material displayed promising performance in matter of microgap and load-bearing capacity.

## References

[1] Barcellos RR, Correia DP, Farina AP, Mesquita MF, Ferraz CC, Cecchin D (2013). Fracture resistance of endodontically treated teeth restored with intra-radicular post: the effects of post system and dentine thickness. J Biomech.

[2] Al-Omiri MK, Mahmoud AA, Rayyan MR, Abu-Hammad O (2010). Fracture resistance of teeth restored with post-retained restorations: an overview. J Endod.

[3] Reeh ES, Messer HH, Douglas WH (1989). Reduction in tooth stiffness as a result of endodontic and restorative procedures. J Endod.

[4] Taha NA, Palamara JE, Messer HH (2011). Fracture strength and fracture patterns of root filled teeth restored with direct resin restorations. J Dent.

[5] Sedgley CM, Messer HH (1992). Are endodontically treated teeth more brittle?. J Endod.

[6] Zarow M, Ramírez-Sebastià A, Paolone G, de Ribot Porta J, Mora J, Espona J, Durán-Sindreu F, Roig M (2018). A new classification system for the restoration of root filled teeth. Int Endod J.

[7] Mohammadi N, Kahnamoii MA, Yeganeh PK, Navimipour EJ (2009). Effect of fiber post and cusp coverage on fracture resistance of endodontically treated maxillary premolars directly restored with composite resin. J Endod.

[8] Yamada Y, Tsubota Y, Fukushima S (2004). Effect of restoration method on fracture resistance of endodontically treated maxillary premolars. Int J Prosthodont.

[9] Sorrentino R, Di Mauro MI, Ferrari M, Leone R, Zarone F (2016). Complications of endodontically treated teeth restored with fiber posts and single crowns or fixed dental prostheses—a systematic review. Clin Oral Investig.

[10] Oskoee PA, Ajami AA, Navimipour EJ, Oskoee SS, Sadjadi J (2009). The effect of three composite fiber insertion techniques on fracture resistance of root-filled teeth. J Endod.

[11] Rocca GT, Krejci I (2013). Crown and post-free adhesive restorations for endodontically treated posterior teeth: from direct composite to endocrowns. Eur J Esthet Dent.

[12] Tay FR, Pashley DH (2007). Monoblocks in root canals: a hypothetical or a tangible goal. J Endod.

[13] El-Helali R, Dowling AH, McGinley EL (2013). Influence of resin-based composite restoration technique and endodontic access on cuspal deflection and cervical microleakage scores. J Dent.

[14] Reeh ES, Douglas WH, Messer HH (1989). Stiffness of endodontically-treated teeth related to restoration technique. J Dent Res.

[15] Wu Y, Cathro P, Marino V (2010). Fracture resistance and pattern of the upper premolars with obturated canals and restored endodontic occlusal access cavities. J Biomed Res.

[16] el-Badrawy WA (1999). Oper Dent.

[17] Zavattini A, Feitosa VP, Mannocci F, Foschi F, Babbar A, Luzi A, Ottria L, Mangani F, Casula I, Sauro S (2014). Bonding ability of experimental resin-based materials containing (ion-releasing)-microfillers applied on water-wet or ethanol-wet root canal dentine. Int J Adhes Adhes.

[18] Ferrari M, Cagidiaco MC, Goracci C, Vichi A, Mason PN, Radovic I (2007). Long-term retrospective study of the clinical performance of fibre posts. Am J Dent.

[19] Seow LL, Toh CG, Wilson NH (2015). Strain measurements and fracture resistance of endodontically treated premolars restored with all-ceramic restorations. J Dent.

[20] Scotti N, Scansetti M, Rota R, Pera F, Pasqualini D, Berutti E (2011). The effect of the post length and cusp coverage on the cycling and static load of endodontically treated maxillary premolars. Clin Oral Investig.

[21] Nothdurft FP, Seidel E, Gebhart F, Naumann M, Motter PJ, Pospiech PR (2008). The fracture behavior of premolar teeth with class II cavities restored by both direct composite restorations and endodontic post systems. J Dent.

[22] Qualtrough AJ, Mannocci F (2003). Tooth-colored post systems: a review. Oper Dent.

[23] Soares CJ, Soares PV, de Freitas Santos-Filho PC (2008). The influence of cavity design and glass fiber posts on biomechanical behavior of endodontically treated premolars. J Endod.

[24] Trope M, Maltz DO, Tronstad L (1985). Resistance to fracture of restored endodontically treated teeth. Dent Traumatol.

[25] Zicari F, Van Meerbeek B, Scotti R (2012). Effect of fiber post length and adhesive strategy on fracture resistance of endodontically treated teeth after fatigue loading. J Dent.

[26] Fráter M, Forster A, Jantyik Á, Braunitzer G, Nagy K, Grandini S (2017). In vitro fracture resistance of premolar teeth restored with fibre-reinforced composite posts using a single or a multi-post technique. Aust Endod J.

[27] Tanner J, Le Bell-Rönnlöf AM, Vallittu PK (2017) Root canal anchoring systems. Chapter 7. In: Vallittu PK, Özcan M (eds) Clinical guide to principles of fiber-reinforced composites in dentistry. Woodhead Publishing, pp 97–108 ISBN 978-0-08-100607-8

[28] Le Bell-Rönnlöf AM, Lassila LV, Kangasniemi I, Vallittu PK (2011). Load-bearing capacity of human incisor restored with various fiber-reinforced composite posts. Dent Mater.

[29] Garoushi S, Vallittu PK, Lassila LV (2007). Direct restoration of severely damaged incisors using short fiber-reinforced composite resin. J Dent.

[30] Forster A, Sáry T, Braunitzer G, Fráter M (2016). In vitro fracture resistance of endodontically treated premolar teeth restored with a direct layered fiber-reinforced composite post and core. J Adhes Sci Technol.

[31] Schneider SW (1971). A comparison of canal preparations in straight and curved root canals. Oral Surg Oral Med Oral Pathol.

[32] Hatta M, Shinya A, Vallittu PK, Shinya A, Lassila LV (2011). High volume individual fibre post versus low volume fibre post: the fracture load of the restored tooth. J Dent.

[33] Scotti N, Coero Borga FA, Alovisi M, Rota R, Pasqualini D, Berutti E (2012). Is fracture resistance of endodontically treated mandibular molars restored with indirect onlay composite restorations influenced by fiber post insertion?. J Dent.

[34] Vallittu PK (1995). Impregnation of glass fibres with polymethylmethacrylate by using a powder coating method. Appl Compos Mater.

[35] Akman S, Akman M, Eskitascioglu G, Belli S (2011). Influence of several fibre-reinforced composite restoration techniques on cusp movement and fracture strength of molar teeth. Int Endod J.

[36] Nicola S, Alberto F, Riccardo MT, Allegra C, Massimo SC, Damiano P, Mario A, Elio B (2016). Effects of fiber-glass-reinforced composite restorations on fracture resistance and failure mode of endodontically treated molars. J Dent.

[37] Soares PV, Santos-Filho PC, Martins LR, Soares CJ (2008). Influence of restorative technique on the biomechanical behavior of endodontically treated maxillary premolars. Part I: fracture resistance and fracture mode. J Prosthet Dent.

[38] Hood JA (1991). Biomechanics of the intact, prepared and restored tooth: some clinical implications. Int Dent J.

[39] Lee MR, Cho BH, Son HH, Um CM, Lee IB (2007). Influence of cavity dimension and restoration methods on the cusp deflection of premolars in composite restoration. Dent Mater.

[40] Lassila L, Keulemans F, Säilynoja E, Vallittu PK, Garoushi S (2018). Mechanical properties and fracture behavior of flowable fiber reinforced composite restorations. Dent Mater.

[41] Manhart J, Chen H, Hamm G, Hickel R (2004). Buonocore Memorial Lecture. Review of the clinical survival of direct and indirect restorations in posterior teeth of the permanent dentition. Oper Dent.

[42] Forster A, Braunitzer G, Tóth M, Szabó BP, Fráter M (2019) In vitro fracture resistance of adhesively restored molar teeth with different MOD cavity dimensions. J Prosthodont 28(1):e325–e331 10.1111/jopr.12777 29508474

[43] Eapen AM, Amirtharaj LV, Sanjeev K, Mahalaxmi S (2017). Fracture resistance of endodontically treated teeth restored with 2 different fiber-reinforced composite and 2 conventional composite resin core buildup materials: an in vitro study. J Endod.

[44] Kemaloglu H, Emin Kaval M, Turkun M, Micoogullari Kurt S (2015). Effect of novel restoration techniques on the fracture resistance of teeth treated endodontically: an in vitro study. Dent Mater J.

[45] Siso SH, Hürmüzlü F, Turgut M, Altundaşar E, Serper A, Er K (2007). Fracture resistance of the buccal cusps of root filled maxillary premolar teeth restored with various techniques. Int Endod J.

[46] Aurélio IL, Fraga S, Rippe MP, Valandro LF (2015) Are posts necessary for the restoration of root filled teeth with limited tissue loss? A structured review of laboratory and clinical studies. Int Endod J 49(9):827–835 10.1111/iej.12538 26331486

[47] Meyenberg K (2013). The ideal restoration of endodontically treated teeth—structural and esthetic considerations: a review of the literature and clinical guidelines for the restorative clinician. Eur J Esthet Dent.

[48] Paolone G, Saracinelli M, Devoto W, Putignano A (2013). Esthetic direct restorations in endodontically treated anterior teeth. Eur J Esthet Dent.

[49] Faria-e-Silva AL, Pedrosa-Filho Cde F, Menezes Mde S, Silveira DM, Martins LR (2009). Effect of relining on fiber post retention to root canal. J Appl Oral Sci.

[50] D'Arcangelo C, Cinelli M, De Angelis F, D'Amario M (2007). The effect of resin cement film thickness on the pullout strength of a fiber-reinforced post system. J Prosthet Dent.

[51] Grandini S, Goracci C, Monticelli F, Borracchini A, Ferrari M (2005). SEM evaluation of the cement layer thickness after luting two different posts. J Adhes Dent.

[52] Garoushi S, Mangoush E, Vallittu P, Lassila L (2013). Short fiber reinforced composite: a new alternative for direct onlay restorations. Open Dent J.

[53] Fennis WM, Tezvergil A, Kuijs RH, Lassila LV, Kreulen CM, Creugers NH, Vallittu PK (2005). In vitro fracture resistance of fiber reinforced cusp-replacing composite restorations. Dent Mater.

[54] Garoushi S, Säilynoja E, Vallittu PK, Lassila L (2013). Physical properties and depth of cure of a new short fiber reinforced composite. Dent Mater.

[55] Fráter M, Forster A, Keresztúri M, Braunitzer G, Nagy K (2014). In vitro fracture resistance of molar teeth restored with a short fibre-reinforced composite material. J Dent.

[56] Chieruzzi M, Pagano S, Pennacchi M, Lombardo G, D'Errico P, Kenny JM (2012). Compressive and flexural behaviour of fibre reinforced endodontic posts. J Dent.

[57] Seefeld F, Wenz HJ, Ludwig K, Kern M (2007). Resistance to fracture and structural characteristics of different fiber reinforced post systems. Dent Mater.

[58] Le Bell AM, Lassila LV, Kangasniemi I, Vallittu PK (2005). Bonding of fibre-reinforced composite post to root canal dentin. J Dent.

[59] Mannocci F, Sherriff M, Watson TF, Vallittu PK (2005). Penetration of bonding resins into fibre-reinforced composite posts: a confocal microscopic study. Int Endod J.

[60] Bitter K, Noetzel J, Neumann K, Kielbassa AM (2007). Effect of silanization on bond strengths of fiber posts to various resin cements. Quintessence Int.

[61] Guzy GE, Nicholls JI (1979). In vitro comparison of intact endodontically treated teeth with and without endo-post reinforcement. J Prosthet Dent.

[62] Wang J, Crouch SL, Mogilevskaya SG (2006). Numerical modeling of the elastic behaviour of fiber-reinforced composites within homogeneous interphases. Compos Sci Technol.

[63] Turkaslan S, Bagis B, Akan E, Mutluay MM, Vallittu PK (2015). Fracture strengths of chair-side-generated veneers cemented with glass fibers. Niger J Clin Pract.

[64] Belli S, Eraslan O, Eskitascioglu G, Karbhari V (2011). Monoblocks in root canals: a finite elemental stress analysis study. Int Endod J.

[65] Garoushi S, Gargoum A, Vallittu PK, Lassila L (2018). Short fiber-reinforced composite restorations: a review of the current literature. J Investig Clin Dent.

[66] Li X, Pongprueksa P, Van Meerbeek B, De Munck J (2015). Curing profile of bulk-fill resin-based composites. J Dent.

[67] Vallittu PK (2016). Are we misusing fiber posts? Guest editorial. Dent Mater.

[68] Patel P, Shah M, Agrawal N, Desai P, Tailor K, Patel K (2016). Comparative evaluation of microleakage of class II cavities restored with different bulk fill composite restorative systems: an in vitro study. J Res Adv Dent.

[69] Aksornmuang J, Nakajima M, Senawongse P, Tagami J (2011). Effects of C-factor and resin volume on the bonding to root canal with and without fibre post insertion. J Dent.

[70] Albashaireh ZS, Ghazal M, Kern M (2010). Effects of endodontic post surface treatment, dentin conditioning, and artificial aging on the retention of glass fiber-reinforced composite resin posts. J Prosthet Dent.

[71] Rocca GT, Saratti CM, Cattani-Lorente M, Feilzer AJ, Scherrer S, Krejci I (2015). The effect of a fiber reinforced cavity configuration on load bearing capacity and failure mode of endodontically treated molars restored with CAD/CAM resin composite overlay restorations. J Dent.

[72] Omran TA, Garoushi S, Abdulmajeed AA, Lassila LV, Vallittu PK (2017). Influence of increment thickness on dentin bond strength and light transmission of composite base materials. Clin Oral Investig.

[73] van Dijken JW, Sunnegårdh-Grönberg K (2006). Fiber-reinforced packable resin composites in class II cavities. J Dent.

[74] Petersen RC (2005). Discontinuous fiber-reinforced composites above critical length. J Dent Res.

[75] Vallittu PK, Lassila VP, Lappalainen R (1994). Transverse strength and fatigue of denture acrylic-glass fiber composite. Dent Mater.

[76] Lazari PC, de Carvalho MA, Del Bel Cury AA, Magne P (2018). Survival of extensively damaged endodontically treated incisors restored with different types of posts-and-core foundation restoration material. J Prosthet Dent.

[77] Wandscher VF, Bergoli CD, Limberger IF, Ardenghi TM, Valandro LF (2014). Preliminary results of the survival and fracture load of roots restored with intracanal posts: weakened vs nonweakened roots. Oper Dent.

[78] Garoushi S, Lassila LVJ, Tezvergil A, Vallittu PK (2007). Static and fatigue compression test for particulate filler composite resin with fiber-reinforced composite substructure. Dent Mater.

